# Mapping of outdoor food and beverage advertising around primary and secondary schools in Kampala city, Uganda

**DOI:** 10.1186/s12889-021-10661-8

**Published:** 2021-04-12

**Authors:** Oumy Erica Wie Dia, Anne Lene Løvhaug, Peter Milton Rukundo, Liv Elin Torheim

**Affiliations:** 1 Department of Nursing and Health Promotion, OsloMet – Oslo Metropolitan University, P.O. Box 4, St. Olavs plass, 0130 Oslo, Norway; 2 Department of Human Nutrition and Home Economics, Kyambogo University, P.O. Box 1, Kyambogo, Uganda

**Keywords:** Food marketing, Outdoor food advertising, Unhealthy foods, Sugar-sweetened beverages, Alcoholic beverages, Primary and secondary schools

## Abstract

**Background:**

Marketing of unhealthy foods and beverages is recognized as a contributing factor to the global increase in overweight and obesity, particularly among children. Such marketing negatively affects children’s dietary preferences, food choices, purchasing requests, and consumption patterns. Given that little is known about food marketing in Africa, including in Uganda, monitoring children’s exposure to food marketing is essential to generate evidence on the problem and develop meaningful policy responses. The aim of this study was to describe the food and beverage marketing environment surrounding schools in urban and peri-urban areas of Kampala city.

**Methods:**

Outdoor advertising around 25 randomly sampled primary and secondary schools within a radius of 250 m of each school was mapped. Information on size, setting, type, and position of the advertisements and the healthiness of the foods and beverages promoted was collected using the INFORMAS Outdoor Advertising Protocol. The occurrence of advertising was described using frequencies, median, and density per 100m^2^.

**Results:**

A total of 1034 branded advertisements were identified around the schools. Of these, 86% featured unhealthy products, 7% healthy products, and 7% miscellaneous products. The most advertised products were sugar-sweetened beverages and alcoholic beverages (51 and 23%, respectively). Schools in the urban area were surrounded by more unhealthy ads than those in the peri-urban areas (median of 45 vs 24 advertisements).

**Conclusion:**

The widespread extent of unhealthy food and beverage advertisements around primary and secondary schools highlights the need for food marketing regulation in Uganda, in line with the World Health Organization’s recommendations, to ensure that young people are protected from unhealthy food marketing.

**Supplementary Information:**

The online version contains supplementary material available at 10.1186/s12889-021-10661-8.

## Introduction

Childhood obesity has become one of the most pressing public health concerns of the twenty-first century [1]. Overweight in children under five is rising in most of the regions of the world [2], exposing them to a greater risk of obesity and diet-related non-communicable diseases as young adults [3]. There is a particular increase in overweight and obesity among school-aged children between 5 and 19 years, with the prevalence having increased tenfold in the last four decades and most rapidly in urban settings in low- and middle-income countries [1, 4, 5].

Among the contributing factors to the global increase in childhood obesity is the marketing of energy-dense, nutrient-poor foods and beverages [6–9]. Children represent a key target for the marketing of food and beverage products [10]. It is well known that marketing of unhealthy foods and beverages (i.e. foods high in sugar, salt, saturated fat and/or trans-fat) can affect children’s dietary preferences, purchasing requests and consumption patterns [6–9], and consequently encourage unhealthy dietary practices [11] which in turn can contribute to rapid weight gain in early childhood [12]. Growing evidence suggests that the vulnerability to, and negative impact of, unhealthy food marketing is not limited to young children, but extends to adolescents [13]. A global study benchmarked children’s exposure to television advertising of unhealthy foods and beverages across 22 countries and found that children are exposed to a large volume of television advertising of unhealthy foods [14]. Similarly, studies have consistently shown that most outdoor food advertisements (ads) are featuring unhealthy foods and beverages [12, 15–18]. Examples of such ads are branded billboards, posters or banners (free-standing/attached, painted or digital), and store merchandise. However, information on the prevalence of outdoor food and beverage advertising in low- and middle-income countries is still limited [9].

During the last 30 years, food companies have increasingly targeted markets in low- and middle-income countries, resulting in the introduction of highly processed foods to the domestic food supply [19–21]. Food marketing is one underlying driver of the worldwide nutrition transition characterized by a shift in dietary patterns from hitherto low fat, low sugar, and high fiber diets to diets that are rich in saturated fat, refined sugars, and low in fiber [22]. Correspondingly, several low- and middle-income countries, including Uganda, are fighting with persistent problems of undernutrition, stunting, and wasting while concurrently experiencing a rapid rise in overweight, obesity, and diet-related non-communicable diseases [23].

The increase in overweight and obesity among children and adolescents in sub-Saharan Africa has been described as alarming but varying from country to country [24]. While there is limited data on the national prevalence of overweight and obesity among school-aged children in Uganda [25–29], modelled estimates indicate a steep increase in overweight among children aged 5–19 years during the timeframe 2001 to 2016 [30, 31].

Given that Uganda has been classified as in the early stage of nutrition transition [32], there may be a “window of opportunity” to implement internationally agreed, knowledge-based prevention measures that support healthy diets at the population level, in particular among children. In the context of food marketing, such measures have been recognized at the World Health Assembly, with Member States having agreed on a set of non-binding recommendations to restrict such unhealthy food and beverage marketing to children [33].

The World Health Organization (WHO) has recommended that national governments should monitor children’s exposure to, and the persuasive power of, food and beverage marketing messages. The *exposure* is defined as the reach and frequency of the message, while *power* is defined as the creative content, design, and execution of the marketing message [33, 34]. Furthermore, the WHO underscores the importance of developing a consistent system for monitoring food and beverage marketing within a country over time and enabling comparison between countries. Such monitoring is essential to determine appropriate and effective policy responses both in a global, national, and local perspective [33].

In light of the evidence linking marketing of products that are high in fat, salt and sugar to childhood obesity, there is also an increasing recognition that countries should, as part of their duties under international human rights law [35, 36], restrict unhealthy food marketing to reduce its negative impact on children, and to realize their rights to health and to adequate food [13, 37].

In Uganda, whereas there are relevant policies on food and nutrition in place, there are presently no specific policy and regulations being implemented with regard to regulating food and beverage marketing. There is also no mechanism in place to monitor and safeguard children’s exposure to food and beverage marketing. Given this knowledge gap, we aimed to map the outdoor food and beverage advertising environment, in terms of extent and power, around selected schools in the capital of Uganda, Kampala.

## Methods

### Study design and setting

The study was carried out in two out of five divisions of Kampala, namely Kampala Central Division and Kawempe Division. Data was obtained in late 2018. The methodology of the International Network for Food and Obesity/non-communicable diseases Research, Monitoring and Action Support framework (INFORMAS) which has been used for similar studies, was applied to investigate the extent and power of the marketing of unhealthy foods and beverages to children. INFORMAS is an independent entity whose work is complementary to monitoring efforts of the WHO, to strengthen the accountability systems needed to help reduce the burden of obesity, NCDs and their related inequalities [38, 39]. The framework provides ten modules including one for monitoring and benchmarking food and beverage promotion [38, 39]. The Outdoor Advertising Protocol of the INFORMAS’ module on food promotion [40] guided the conceptualization of this study. The study was part of a larger project which also included qualitative approaches to explore national policymakers’ perspectives on unhealthy food marketing; the qualitative part will be reported in a separate publication.

### Sampled sites

This study included 13 primary schools and 12 secondary schools which covered children in the age group 6–19 years. Considering the number of schools that have been included in previous similar studies in high-income [17] and low- and middle income [41, 42] countries, and taking into account the explorative nature of the study, twenty-five schools were deemed to be a reasonable sample size. Since the term ‘child’ covers all children and adolescents under the age of 18 years [3, 33, 43]**,** it was essential to include both primary and secondary schools in this study. Both day schools, and mixed day and boarding schools were included. Boarding schools were excluded since children attending these schools were encamped and not allowed to go outside the school area during the semester; thus, they were not usually exposed to outdoor food marketing.

The sample of schools was selected through a multi-stage sampling approach following the Outdoor Advertising Protocol [40]. In the first stage of sampling, the most urban division, and a less urban [hereinafter called peri-urban] division of Kampala were selected: Kampala Central Division and Kawempe Division, respectively. Kampala Central is the smallest division in Kampala District, located in an urban area, the city center, while Kawempe is the largest division, located in the Northern part of Kampala District, and is a more peri-urban area with a lower population density and income level than Kampala Central. Separate lists of primary and secondary schools in each of the two divisions were generated following a mapping and listing exercise using information available from the Directorate of Education and Social Services at Kampala Capital City Authority (KCCA). In the second stage of the sampling, 7 out of 25 primary schools in Kampala Central, and 6 out of 56 primary schools in Kawempe were selected from the lists of primary schools using the simple random number generator in Microsoft Excel. The same process was followed for secondary schools where 6 out of 8 secondary schools in Kampala Central and 6 out of 25 secondary schools in Kawempe were randomly selected. Of the sampled schools, however, nine were either permanently closed, not locatable, or located outside the division boarder. Consequently, an equal number of schools were replaced by selecting the following school on the respective list. The study involved no human subjects.

### Data collection

For each school, an electronic map was generated with circles to indicate a radius of 250 m from the entrance/boundary of the school, with the use of Map Developers [44]. The radius of 250 m around the schools is in accordance with the INFORMAS protocol [40], and has been used in other comparable studies [17, 41]. These studies have however also included an additional 500 m radius around schools, whereas a study from Mexico used a 100 m radius [42].

The maps were printed and used manually during the data collection. The Outdoor Advertising Protocol’s standard template [40] was used to record the ads. For each advertisement, information was collected on:

i) The distance of the food/beverage advertisement from school (within 250 m).
ii) The size of the advertisement (small (21 cm × 30 cm - 1.3 m × 1.9 m), medium (> 1.3 m × 1.9 m - 2.0 m × 2.4 m), large (≥ above 2 m × 2.5 m).
iii) The setting of the advertisement (food shop, roads, building, bus shelter, train station, cart/stall).
iv) The type and position of the advertisement (billboard, poster, free-standing, painted, digital/LED, store merchandising).
v) Whether the subject of the advertisement was for single or multiple foods and beverages.
vi) The food/beverage brand name(s) and product type(s).
vii) Major food category (core/healthy, non-core/unhealthy, miscellaneous).
viii) Minor food category (e.g. sugar-sweetened beverages, alcoholic beverages, savoury food snacks, healthy food snacks, water, baby foods, baby and toddler milk), divided into 37 food categories [40].
ix) Any promotional character and premium offers.

Three locally recruited public health nutritionists with at least a bachelor’s degree qualification were hired and trained to assist in the data collection processtogether with the first author (EWD). The data was recorded manually, and each sample site was visited once. The school zones were cross-checked to ensure that all ads in every street and corner were included. The data collection was completed within two weeks, to ensure that all ads were recorded in the same time period, and thus, limiting the risk of any seasonal fluctuations that occur in advertising frequency and power of exposure over a year cycle.

### Coding

The ads were coded by the first author (EWD) on the day of data collection. An ‘advertisement’ was defined as a sign with branded information, pictures, or logos for food or beverage products or companies. This included billboards, posters, free-standing signs, neon signs, stickers, electronic boards, banners, bus shelter signs and signs on outdoor furniture, bridge/awning signs, and painted buildings. Store signages that also had a product logo and served not just as a store identifier but also as promotional material for a product were considered ads. Ads smaller than A4 size, signage used mainly for store identification, and pictures of unbranded restaurant foods or other foods were excluded from the study. All A-frame double-sided bus shelters and standing signs where ads appeared on two sides with different content on each side were identified and coded separately to ensure that accurate information was collected.

An advertisement was considered ‘unhealthy’ when at least one food product in the advertisement was categorized as unhealthy following the Outdoor Advertising Protocol and the WHO nutrient profiling model [45, 46].

### Data analyses

Data was imported into IBM SPSS for Windows version 24 (SPSS Inc., Chicago, IL.). The schools were divided into tertiles of school fee, as an indicator of the school’s socio-economic status. Additionally, the schools were classified into government-funded schools and private schools to serve as another proxy of socio-economic status. The rationale behind this was that private schools have fee structures that some families from low-income groups may not fully afford, while government-funded schools have lower school fees [47].

The density of ads within a radius of 250 m (school zone) was calculated (ads per 100 m^2^). For each site, descriptive analyses were conducted to determine the frequency, median, and density of food ads by product type and content, setting and size, school areas (divisions), school types (primary and secondary), school fees (low, medium, and high) and school categories (government-funded and private). Non-parametric tests were used since the data was not normally distributed [48]. Mann-Whitney U test was used to compare two independent groups, and Kruskal-Wallis test to compare three independent groups. The statistical significance level was set at *α <* 0.05 for all analyses.

Henceforth, the term “food” in this paper, is used to refer to foods and beverages.

## Results

### Characteristics of the study population

The sample consisted of 13 primary schools and 12 secondary schools. Table 1 presents the total number of schools by type, category, fee structure respectively, and the number of schools distributed by urban and peri-urban areas.

**Table 1:** Characteristics of the study units

	Total schools	Schools in Kampala Central Division (urban)	Schools in Kawempe Division (peri-urban)
School characteristics	n	n	n
School type			
Primary schools	13	7	6
Secondary schools	12	6	6
School category			
Government-funded schools	9	8	1
Private schools	16	5	11
School fee			
Low school fee	9	6	3
Medium school fee	8	4	4
High school fee	8	3	5
Total	25	13	12

### Description of the advertised foods and beverages

The study mapped 1034 branded ads around 25 schools (Table 2). Most of the adverts were for unhealthy foods and beverages (86%), 7% were for healthy foods and 7% were for miscellaneous foods (tea/coffee/condiments). The most frequently advertised food products were sugar-sweetened beverages (51% of all ads), followed by alcoholic beverages (23%) and high fat and/or sugar flavoured dairy products (5%). The number of ads varied between the schools, of which the majority ranged between 20 to 70 adverts (Supplementary Table 1, Additional file 1). There was an almost absence of infant formula advertising (*n* = 9, 1% of the total ads (Table 2).

**Table 2:** Type and frequency of promoted foods and beverages around the schools (% of total ads)

Major and minor food categories^**a**^	Number	Percent
**Healthy foods**	**85**	**7**
Bottled water	31	3
Healthy oils and low-fat savory sauces	21	2
Staple foods/plain starch products	12	1
Low-fat dairy and dairy alternatives, and drinks	11	1
Meat and meat alternatives	4	0
Low sugar, high fiber cereals	2	0
Fruit/fruits products without added sugar	2	0
Healthy snacks	2	0
**Unhealthy foods**	**887**	**86**
Sugar-sweetened beverages	522	51
Alcohol	233	23
High fat and/or sugar flavoured dairy products	56	5
Chocolate and candy	25	2
Fast food	15	2
Savoury snack food	9	1
Other high fat/salt products	9	1
Sweet breads, biscuits, pies, and pastries	6	1
Ice cream and desserts	3	0
Fruit juice/drinks (< 98%)	3	0
Sugar-rich, low fiber cereals	3	0
Ultra-processed meat and meat alternatives	2	0
**Miscellaneous**	**62**	**7**
Condiments, seasonings and recipe additions	47	5
Baby and toddler milk formulae	9	1
Vitamin or dietary supplements	4	1
Tea and coffee	2	0
**Total**	**1034**	**100**

^a^ Major food categories (in bold): healthy/unhealthy/miscellaneous. Minor food categories: the type of food product advertised under their respective major food category

### Types of advertised foods and beverages

**Fig. 1:**
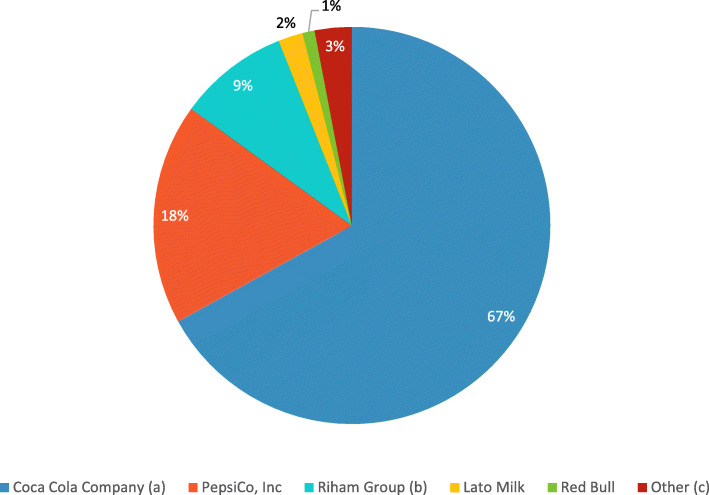
The proportion of sugar-sweetened beverages by advertising companies. ^a^ Excluding Coca-Cola ads promoted with fast foods. Instead, the nine ads where Coca Cola and fast food was combined in one ad were coded as minor food category 22 titled “fast food”. ^b^ Riham Group under Hariss International Limited. ^c^ Companies that in total accounted for less than 1 % of the sugar-sweetened advertisements fell under the category titled “other”

Eighty-eight percent of the schools were predominantly exposed to sugar-sweetened beverage ads, followed by alcoholic beverage ads (Supplementary Table 2, Additional file 1). A total of 115 different companies were identified as advertisers in the study. Of these companies, Coca-Cola Company, PepsiCo Ink, and Uganda Breweries Ltd. accounted for 35%, 9%, and 9% of the ads surrounding schools, respectively. Within the sugar-sweetened beverages category, Coca Cola accounted for 67% of the ads (Fig. 1).

### Food and beverage advertisements by setting and display type

In terms of setting, half of the ads were on buildings (51%), followed by food shops (32%) and roads (13%). Less commonly marketing settings were mobile charts, stalls or vending machines (4%) and bus shelters (2 ads). By display type, almost two-thirds of the ads were posters or banners (66%), followed at by-store merchandising (15%), free-standing signs/signage (11%), painted building/wall (6%), billboards and digital signs or LED (1% in both).

### Food and beverage advertisements by urban and peri-urban areas

**Fig. 2:**
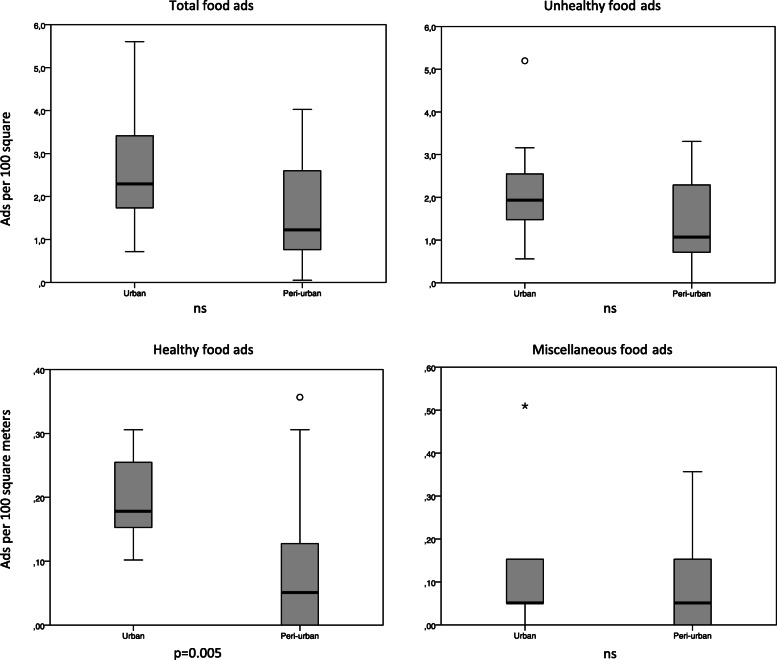
Density (per 100 m^2^) of food and beverage advertisements (total and by major food categories) by urban and peri-urban areas

The number of ads was higher around schools in the urban area compared to the peri-urban area, with a median of 45 and 24, respectively (Table 3), but the difference was not significant. This translated to an overall density of ads of 2.6/100 m^2^ in the urban and 1.6/100 m^2^ in the peri-urban area (Fig. 2) (Supplementary Table 1, Additional file 1). In terms of unhealthy ads, there was no significant difference between the respective areas. Although healthy ads were infrequently observed, the number of healthy ads was significantly higher around schools in the urban area compared to the peri-urban area (4 vs. 1, *p* = 0.005, Table 3).

**Table 3:** Median (25-, 75-percentiles) number of food and beverage advertisements (total and by major food categories) within a radius of 250 m around the school, by school characteristics (*n* = 25)

	Total food ads	Major food categories		
*School characteristics*		Unhealthy	Healthy	Miscellaneous
*School area*				
Urban areas (*n* = 13)	45 (34, 67)	38 (29, 50)	4 (3, 5)	1 (1, 3)
Peri-urban areas (*n* = 12)	24 (15, 51)	21 (14, 45)	1 (0, 3)	1 (0, 3)
*p*-value ^a^	0.077	0.11	0.005	0.73
*School fee level*				
Low (*n* = 9)	45 (33, 53)	36 (29, 45)	4 (3, 5)	1 (1, 7)
Medium (*n* = 8)	44 (22, 63)	38 (21, 55)	3 (1, 5)	1 (1, 3)
High (*n* = 8)	33 (22, 45)	31 (19, 42)	2 (1, 3)	1 (0, 3)
p-value ^b^	0.69	0.75	0.33	0.41
*School type*				
Primary (*n* = 13)	41 (33, 58)	37 (29, 45)	3 (1, 5)	2 (1, 7)
Secondary (*n* = 12)	30 (17, 53)	27 (15, 50)	2 (1, 4)	1 (0, 3)
p-value ^a^	0.29	0.47	0.27	0.25
*School category*				
Government-funded (*n* = 9)	45 (33, 67)	39 (29, 50)	4 (3, 5)	1 (1, 3)
Private (*n* = 16)	38 (22, 52)	33 (19, 45)	2 (1, 4)	2 (0, 3)
p-value ^a^	0.56	0.56	0.084	0.85
Total	40 (22, 55)	36 (19, 47)	3 (1, 5)	1 (0,3)

^a^ Differences between groups measured with Mann-Whitney U test

^b^ Differences between groups measured with Kruskal Wallis test

### Food and beverage advertisements by school type, school fee, and school category

There were no significant differences in the median number of unhealthy or healthy ads around the different types of schools when comparing primary schools versus secondary schools; schools with low, medium, and high school fees; or between government-funded and private schools (Table 3) (Supplementary Table 1, Additional file 1).

### Size of food and beverage advertisements

When ranging the size of the ads, small or medium ads were most frequently occurring around schools, while large size comprised of approximately a quarter of the ads (40, 38, 22%, respectively). There was no association between the size of the ads and the proportion of unhealthy ads (Table 4).

**Table 4:** Food advertising (total and by major food categories) by the size of the advertisement and by use of promotional characters (*n* = 1034)

	Total food ads *N* = 1034		Major food categories						
			Unhealthy *n* = 889		Healthy *n* = 84		Miscellaneous *n* = 61		p-value^a^
	n	%	n	%	n	%	n	%	
*Advertisement size*									n.s.
Small ad	409	39.6	351	39.5	34	40.5	24	39.4	
Medium ad	396	38.3	340	38.2	30	36.9	25	38.3	
Large ad	229	22.1	198	22.3	19	22.6	12	22.1	
*Promotional characters*									0.002
No	957	92.6	825	92.8	71	84.5	61	100	
Yes	77	7.4	64	7.2	13	15.5	0	0	

^a^ Pearson’s Chi square

### The power of promotion

Of all the ads (*n* = 1034), 7.4% included promotional characters (*n* = 77) (Table 4). Promotional characters were found in a higher proportion of the healthy food ads compared with the unhealthy food ads (15% vs 7%, *p* = 0.002). The promotional characters most used were cartoons or company-owned characters (74%), followed by an unknown character (9%), licensed characters (8%), ‘for kids’ images and messages (7%), and famous sportspersons, celebrities, and events (1% in all). Premium offers were less commonly used and only found in unhealthy ads (*n* = 13, 1.3%).

## Discussion

The results demonstrate a total of 1034 food ads around 25 randomly selected schools in two divisions in Kampala. There were on average 40 ads within a radius of 250 m around each of the schools. Of these, a large majority (86%) were for unhealthy foods with an average of 36 unhealthy food ads around each school. Whereas there is a scarcity of previous comparable studies from low-income countries, Kelly et al. [41] documented a lower number of ads around schools in Ulaanbaatar in Mongolia (mean of 18 ads), whereas a higher number (128 ads) was documented around schools in Manila, the Philippines [41]. In line with the present study, unhealthy foods accounted for the majority of the ads in both settings (92% in Ulaanbaatar and 85% in Manila).

Our findings are consistent with research in a high-income country (Australia) that documented ads within a 500 m radius of forty primary schools in Sydney and Wollongong. Out of a total of 2287 food ads, 80% were for unhealthy foods, and the density was twice as high in the area closest to schools (≤ 250 m) [17]. The increasing number of studies that monitor food advertising thus indicate that unhealthy food advertising is a public health challenge, particularly in urban areas in both low-, middle-, and high-income countries [15, 17, 41, 42, 49]. Particularly for the low- and middle-income countries, it has been postulated that food marketing, in the form of advertising and product placement in communities that otherwise have limited access to these food items, may potentially accelerate the nutrition transition [50]. A recent study explored the urban and rural environments in cities of different income levels, and documented that Uganda had the highest in-community food advertising when compared to South Africa and Sweden [51].

The overall density of ads was higher in urban areas, which has a greater population density and less social disadvantages than peri-urban areas. A similar pattern was observed in Sydney and Wollongong, where there was significantly more advertising in high population density/high socioeconomic status areas [17]. Similarly, the study in Ulaanbaatar found that the overall density of ads was more than twice as high in areas of greater population density and socio-economic status [41].

For the marketing industry, there is value in advertising in high-density areas as more people come in contact with the branded product, thereby increasing the overall brand exposure [17]. Concurrently, urban settings are recognized as more vulnerable to food and lifestyle choices that prioritize the consumption of ‘take away’ and other foods that can be energy-dense and nutrient-poor [52]. In Kampala, a switch from a traditional diet to a diet rich in processed foods high in sugars, salt and fat has been reported [53]. The number of healthy ads surrounding schools in the urban area was significantly higher than in the peri-urban area, reflecting neighbourhood disparities in healthy food promotion. The lower-income areas were scarcely exposed to marketing that promoted healthy eating habits and optimum health, representing a concern that also has been established in previous studies [54–57]. Furthermore, as recognized in The State of Food Security and Nutrition in the World 2019 report, poorer communities often face physical and economic barriers to obtaining nutritious foods, placing them at higher risk of food insecurity and malnutrition [58].

### Exposure to sugar-sweetened beverages

More than half of the ads captured in this study were for sugar-sweetened beverages, followed by alcoholic beverages (51 and 23%, respectively). The findings are in line with previous studies that examined outdoor ads around schools. Kelly et al. (2008) found that sugar-sweetened beverages (24%) and alcoholic beverages (22%) were most frequently advertised in New Zealand. Similarly, in Manila, it was estimated that more than half of the unhealthy ads surrounding schools were for sugar-sweetened beverages (56%), followed by alcoholic beverages (7%) [41].

In the present study, Coca-Cola accounted for 35% of the total ads and 67% of the sugar-sweetened beverages. Studies from Ulaanbaatar and Manila have reported similar findings, showing that Coca-Cola is a highly featured brand [41]. Similarly, research from Accra city in Ghana, which used a different methodology, found that the majority of ads featured sugar-sweetened beverages (73%) and that Coca-Cola accounted for 60% of the total ads [59]. The present study identified that two of the schools had large school signs branded by Coca-Cola at their school entrance but cannot confirm whether the schools received any monetary or program support from the sponsoring company. In Western Cape, however, a study identified that 60% of the schools featured a signage board with the school’s name and a branded soft drink, of which 85% were sponsored by a well-known brand [60]. Such ads may imply that the schools are endorsing the marketing message [13]. This type of advertising is in breach with WHO recommendations [33] and indicates the urgent need for national regulations of unhealthy food advertising.

### Exposure to alcoholic beverages

The fact that alcoholic beverages was the second most promoted food category around schools (23% of all ads) is of high public health concern. Uganda had an annual per capita alcohol consumption of nearly 24 liters in 2014, which increased to 26 liters in 2016, ranking among the countries with the higher alcohol consumption rates in Africa [61, 62]. A high alcohol consumption rate is also reported to be prevalent among youth in the country [61]. A previous study reported that a sample of schools had not instituted serious measures to prevent the onset of alcohol consumption in schools, nor had the communities where students came from been supportive [63]. Given the harmful effect associated with alcoholic beverages [61, 64] and the age limit of 18 years, such advertising is inconsistent with any health-promoting recommendations. As for unhealthy food, there is an urgent need for regulation of alcohol marketing in Uganda. A complete ban on alcohol advertising has been proposed by both international health organizations [65, 66] and national policy-makers such as Uganda Alcohol Policy Alliance, and by the Parliament of Uganda’s draft on an Alcoholic Drinks Control Bill 2016, reflecting the ongoing momentum for clear policy framework to protect children and the vulnerable populations from both the consumption and effects of alcohol [67].

### Policy enforcement and implementation

The study observed an almost absence of marketing of toddler milk/infant formula indicating that Uganda’s Code of Conduct for breastmilk substitutes is strong and well enforced [68–71]. The findings also suggest that an international food marketing framework similar to the Code of Marketing of Breastmilk Substitutes [72] could perhaps serve as a model for future policies designed to reduce unhealthy food marketing practices in Uganda and other parts of the world.

The need to address the double burden of malnutrition, ensure food safety, and encourage healthy food marketing is recognised in the 2020 annual report of the work of WHO in the African Region, where fifteen West African countries were supported to strengthen food and beverage regulation, including alcoholic beverages [73]. This support embodies the growing recognition of unhealthy food marketing as a major health and children’s rights issue. Acting on food environments by discouraging unhealthy food marketing and simultaneously ensuring healthy diet availability, affordability, and appeal is one opportunity to prevent malnutrition in all its forms [74].

Our findings demonstrate that there is a need to reduce the marketing of unhealthy foods and beverages around schools, and in particular of sugar-sweetened beverages and alcoholic beverages. A ban on marketing of unhealthy foods and beverages within a radius of at least 100 m (as recommended by Pan American Health Organization [75] would be an important step towards healthier food environments in proximity to schools. The implementation of such policies also contributes to meeting state obligations to protect, respect, and fulfil children’s rights under international human rights law.

### Strengths and limitations

This study has verified the possibility of mapping ads comprehensively with the use of an acknowledged and standardized protocol. Its findings provide insights into the advertising environment in school zones of the most densely populated areas in Uganda. The inclusion of a peri-urban area may, to some extent, provide insight into a partly rural context. Importantly, the findings show that it is essential for future studies to include alcoholic beverage ads when exploring food marketing. A limiting factor is that the sample size was set to 25 schools within two out of five divisions of the city. Consequently, the results cannot be generalized to all schools and zones in the larger city*.*

At the time of the study, there was no available nutrient profiling model for the African region. Since then, the nutrient profiling model for the African region has been adopted [76]. In this study, we applied the possibility of mapping food marketing in an African city context using a European-developed framework. As such, there is a risk for having overlooked the nature of food environments in Africa. For example, we excluded what could be a significant proportion of outdoor ads; those that were on the inside of the ‘food shop door’ when closed but faced the outside when open. Based on observation, the majority of these were unhealthy food ads. Also, since we set a 250-m buffer around schools, the result may only serve as a proxy for the area of true exposure relevant to children [18], rather than the total exposure of food advertising near schools.

In addition to the highlighted limitations, there is a lack of comparable studies in the African context which limits the transferability to similar country contexts. Going forward, this study may give a basis for situation-analysis, monitoring, guidance, comparison, and referring purposes. The study may also serve as a starting point for future studies, research agendas and collaborations in this field and other related areas of public health nutrition.

## Conclusion

Overall, the results suggest that the food marketing landscape around schools in Kampala was not conducive to a health-promoting environment in the dimension of food advertising. Repeated exposure to such food marketing may encourage children to consume these foods and beverages, which is recognized as a major contributing factor to unhealthy diets, obesity, and non-communicable diseases.

The findings have particular implications for policies that regulates advertising of sugar-sweetened beverages and alcoholic beverages. Such policies are presently limited in Uganda, yet it is of the essence to have a robust policy and legal agenda to mitigate the malnutrition and disease risk that may accrue from unregulated advertisement of unhealthy foods and beverages. Despite surveillance data limitations, the prevalence of overweight and obesity among children in Uganda is on the rise and the burden seems to be greater in the urban communities, such as Kampala. A disease preventative and health-promoting measure to prevent acceleration of the double burden of malnutrition, could be to raise attention to unhealthy food marketing to children; and to develop relevant marketing policies and comply with such policies once they are enforced in order to foster accountability.

## Supplementary Information

**Additional file 1.**

## Data Availability

The dataset generated and analysed during the current study are available from the first author upon reasonable request and with permission of INFORMAS.
