# Supplementary material for: Mapping of outdoor food and beverage advertising around primary and secondary schools in Kampala city, Uganda
Source: BMC Public Health. 2021 Apr 12;21:707. doi: 10.1186/s12889-021-10661-8 (PMC8042698; doi:10.1186/s12889-021-10661-8)
Supplement: Supplementary file 1 — Additional file 1. [file 12889_2021_10661_MOESM1_ESM.docx]

**Mapping of outdoor food and beverage advertising around primary and secondary schools in Kampala city, Uganda**

Oumy Erica Wie Dia^1^, Anne Lene Løvhaug^1^, Peter Milton Rukundo^2^, Liv Elin Torheim^1^

^1^Department of Nursing and Health Promotion, OsloMet – Oslo Metropolitan University, Norway

^2^Department of Human Nutrition and Home Economics, Kyambogo University, Uganda

Supplementary Table 1. Density (per 100 m^2^) of food and beverage advertisements (total and by major food categories) by school characteristics (n=25), median (25-, 75-percentiles)

|  | Total food ads | Major food categories | | |
| --- | --- | --- | --- | --- |
| *School characteristics* |  | Unhealthy | Healthy | Miscellaneous |
| *School area* |  |  |  |  |
| Urban areas (*n* = 13) | 2.3 (1.7, 3.4) | 1.9 (1.5, 2.5) | 0.2 (0.2, 0.3) | 0.05 (0.05, 0.15) |
| Peri-urban areas (*n* =12) | 1.2 (0.8, 2.6) | 1.1 (0.7, 2.3) | 0.05 (0, 0.1) | 0.05 (0, 0.2) |
| p-values^a^ | 0.077 | 0.11 | 0.005 | 0.73 |
| *School fee level* |  |  |  |  |
| Low (*n* = 9) | 2.3 (1.7, 2.7) | 1.8 (1.5, 2.3) | 0.2 (0.2, 0.3) | 0.05 (0.05, 0.4) |
| Medium (*n* = 8) | 2.2 (1.1, 3.2) | 1.9 (1.1, 2.8) | 0.1 (0.03, 0.3) | 0.1 (0.05, 0.2) |
| High (*n* = 8) | 1.7 (1.1, 2.3) | 1.6 (1.0, 2.1) | 0.1 (0.05, 0.2) | 0.03 (0, 0.2) |
| p-values^b^ | 0.69 | 0.75 | 0.33 | 0.41 |
| *School type* |  |  |  |  |
| Primary (*n* = 13) | 2.1 (1.7, 3.0) | 1.9 (1.5, 2.3) | 0.2 (0.05, 0.3) | 0.1 (0.05, 0.4) |
| Secondary (*n* = 12) | 1.5 (0.9, 2.7) | 1.4 (0.8, 2.5) | 0.10 (0.05, 0.2) | 0.05 (0, 0.2) |
| p-values^a^ | 0.29 | 0.47 | 0.27 | 0.25 |
| *School category* |  |  |  |  |
| Public (*n* = 9) | 2.3 (1.7, 3.4) | 2.0 (1.5, 2.5) | 0.2 (0.2, 0.3) | 0.05 (0.05, 0.2) |
| Private (*n* = 16) | 1.9 (1.1, 2.6) | 1.7 (1.0, 2.3) | 0.1 (0.03, 0.2) | 0.08 (0.0, 0.2) |
| p-values^a^ | 0.56 | 0.56 | 0.084 | 0,85 |
| Total | 2.0 (1.1, 2.8) | 1.8 (1.0, 2.4) | 0.1 (0.05, 0.3) | 0.05 (0, 0.2) |

^a^Differences between groups measured with Mann-Whitney U test

^b^Differences between groups measured with Kruskal Wallis test

Supplementary Table 2. Advertisements per school and by its respective city division

Number and percent by major food category (unhealthy, healthy, miscellaneous) and by minor food category code (the type of food product promoted most frequently promoted around the respective school code, and the respective percentage).

| **Schools Code** | **Total food ads**  n (%)^c^ | **Major food categories** ^d^ | | | | **The most promoted food (and % it accounted for)**^e^ |
| --- | --- | --- | --- | --- | --- | --- |
|  |  | **Unhealthy**  n (%) | **Healthy**  n % | **Miscellaneous**  n (%) | |  |
| **Urban area** |  |  |  | |  |  |
| 1^a^ | 45 (4) | 39 (87) | 5 (11) | | 1 (2) | SSB (62) |
| 2^a^ | 41 (4) | 39 (93) | 3 (7) | | 0 (0) | SSB (46) |
| 3^a^ | 33 (3) | 29 (88) | 3 (9) | | 1 (3) | SSB (55) |
| 4^a^ | 35 (3) | 30 (86) | 3 (9) | | 2 (6) | SSB (54) |
| 5^a^ | 71 (7) | 46 (65) | 15 (21) | | 10 (14) | SSB (24) |
| 6^a^ | 51 (5) | 36 (71) | 4 (9) | | 11 (22) | SSB (55) |
| 7^a^ | 110 (11) | 102 (93) | 5 (4) | | 4 (4) | SSB (53) |
| 8^b^ | 34 (3) | 29 (85) | 4 (12) | | 1 (3) | SSB (56) |
| 9^b^ | 26 (3) | 24 (92) | 2 (8) | | 0 (0) | SSB (42) |
| 10^b^ | 53 (5) | 50 (94) | 3 (6) | | 0 (0) | SSB (53) |
| 11^b^ | 67 (6) | 60 (90) | 5 (7) | | 2 (3) | SSB (52) |
| 12^b^ | 71 (7) | 61 (87) | 7 (10) | | 3 (4) | SSB (48) |
| 13­­­­­^b^ | 14 (1) | 11 (79) | 2 (14) | | 1 (7) | SSB (43) |
| **Peri-urban area** |  |  |  | |  |  |
| 14^a^ | 40 (4) | 37 (93) | 0 (0) | | 3 (7) | SSB (58) |
| 15^a^ | 58 (6) | 45 (78) | 6 (10) | | 7 (12) | SSB (57) |
| 16^a^ | 79 (8) | 65 (82) | 7 (9) | | 7 (9) | SSB (42) |
| 17^a^ | 10 (1) | 9 (90) | 1 (10) | | 0 (0) | SSB (60) |
| 18^a^ | 20 (2) | 19 (95) | 1 (5) | | 0 (0) | SSB (80) |
| 19^a^ | 24 (2) | 23 (96) | 0 (0) | | 1 (4) | AB (54) |
| 20^b^ | 5 (0.5) | 4 (80) | 1 (20) | | 0 (0) | Sweets/SSB (40) |
| 21^b^ | 1 (0.1) | 0 (0) | 0 (0) | | 1 (100) | Condiments (100) |
| 22^b^ | 24 (2) | 19 (79) | 2 (8) | | 3 (13) | SSB (71) |
| 23^b^ | 20 (2) | 19 (95) | 1 (5) | | 0 (0) | SSB (55) |
| 24^b^ | 53 (5) | 50 (94) | 0 (0) | | 3 (6) | AB (43) |
| 25^b^ | 49 (5) | 45 (92) | 3 (6) | | 1 (2) | SSB (63) |
| **Total ads** | **1034 (100)** | **887** | **85** | | **62** | **-** |

^a^ Primary school; ^b^ Secondary school

^c^ Total number of ads in the respective school

^d^ Number (and percentage) of each major food category in the respective school.

^e^ The most promoted minor food category (and the % it accounted for) in the respective school, according to the minor food categories defined in the INFORMAS Outdoor Advertising Protocol ^39^(pp27-28): Candies = minor food category number 21 (chocolate and candy); SSB= minor food category number 25 (sugar-sweetened beverages); AB= minor food category number 26 (alcoholic beverages); Condiments = minor food category number 27 (recipe additions).
